# Core-Shell Heterostructured and Visible-Light-Driven Titanoniobate/TiO_2_ Composite for Boosting Photodegradation Performance

**DOI:** 10.3390/nano9101503

**Published:** 2019-10-22

**Authors:** Chao Liu, Xin Gao, Zitong Han, Yao Sun, Yue Feng, Guiyun Yu, Xinguo Xi, Qinfang Zhang, Zhigang Zou

**Affiliations:** 1 School of Materials Science and Engineering, Yancheng Institute of Technology, Yancheng 224051, China; cliu@ycit.edu.cn (C.L.); gaoxinvv123@163.com (X.G.); zitonghan@hotmail.com (Z.H.); sunyao98@hotmail.com (Y.S.); fengy98@hotmail.com (Y.F.); 2 Eco-Materials and Renewable Energy Research Center (ERERC), College of Engineering and Applied Sciences, Nanjing University, Nanjing 210093, China; zgzou@nju.edu.cn; 3 School of Chemistry & Chemical Engineering, Yancheng Institute of Technology, Yancheng 224051, China; yuguiyun1@163.com; 4 Key Laboratory for Advanced Technology in Environmental Protection of Jiangsu Province, Yancheng Institute of Technology, Yancheng 224051, China

**Keywords:** titanoniobate, core-shell structure, photocatalytic degradation, visible light

## Abstract

Herein, we report a one-dimensional (1D) S-doped K_3_Ti_5_NbO_14_@TiO_2_ (STNT) core-shell heterostructured composite with an enhanced photocatalytic degradation activity under visible light, which was prepared by a simple reassembly-calcination method using thiourea as the S source. The anisotropically shaped rods are favorable for the rapid transport of photogenerated charge carriers. The substitution of Ti^4+^ by S^6+^ is primarily incorporated into the lattice of the TiO_2_ shell so as to create an intra-band-gap state below the conduction band (CB) position, giving rise to Ti−O−S bonds and thus the visible light response. The presence of electron-deficient S atoms is of benefit to the decreased recombination rate of photogenerated electrons and holes by capturing electrons (e^−^). Meanwhile, a tight close interface between K_3_Ti_5_NbO_14_ and TiO_2_ was formed to achieve a nano-heterojunction structure, leading to the fostered separation of its interfacial photogenerated electrons and holes. The visible-light-driven photocatalytic degradation of methylene blue (MB) by STNT composites is higher than that by pure K_3_Ti_5_NbO_14_, owing to the synergistic effects of S doping and heterojunction. A possible photocatalytic mechanism was proposed with a reasonable discussion. This work may provide an insight into constructing highly efficient core-shell photocatalysts used toward sustainable environmental remediation and resource shortages.

## 1. Introduction

With the rapid development of global industrialization and population explosion, organic pollutants show negative impacts on human health and ecosystem equilibrium due to the difficult removal from wastewater [1,2,3]. It is widely accepted that semiconductor photocatalytic technology, as a green method, plays an important role in eliminating most organic contaminants and thus constructing an environmentally-friendly society [4,5]. As a traditional photocatalyst, TiO_2_ has been profoundly investigated due to its advantageous properties, such as good chemical stability, exceptional electronic property, low toxicity, and low cost [6,7]. However, pure TiO_2_ shows some drawbacks, such as it is unresponsive to visible light and it has a quick recombination rate of photogenerated electron–hole pairs [8,9,10]. Therefore, more and more attention has been paid to construct novel materials with high photocatalytic performance, especially under visible light.

Layered titanoniobate, like K_3_Ti_5_NbO_14_, has attracted much attention due to its layered structure, anisotropic rod-like shape, and typical electronic properties [11,12,13]. Especially, anisotropically shaped rods can provide a channel for the rapid transport of charge carriers along the longitudinal direction, leading to the decreased recombination rate of photogenerated electrons and holes [14]. However, wide-band-gap semiconductors of pure K_3_Ti_5_NbO_14_ can only utilize UV light and shows the rapid recombination rate of photogenerated electron–hole pairs. Thus, it is of great significance to employ different modified methods for designing visible-light-driven K_3_Ti_5_NbO_14_-based photocatalysts with high photocatalytic activity.

To enhance the photocatalytic activity of semiconductor catalysts, many approaches, such as elemental doping, heterojunction formation, and morphological design, have been widely employed. Generally, doping semiconductors with non-metals can greatly extend light absorption [15,16,17]. For example, our group developed a series of S-doped TiO_2_-based composites, showing improved photocatalytic activity [18,19]. Constructing a heterojunction has been regarded as an efficient approach to improve spatial charge separation between different semiconductors owing to the interfacial electric field [20,21]. Recently, our group employed a simple calcination method to prepare K_3_Ti_5_NbO_14_/g-C_3_N_4_ composites, showing higher visible-light-driven photodegradation activity than that of its counterparts [22]. The improved photocatalytic activity was ascribed to the synergistic effects of anisotropic K_3_Ti_5_NbO_14_ rod for the high-charge carrier mobility, the layered heterojunction for quick separation of photo-induced electrons and holes, and N-doping for the improvement of the light response region. Furthermore, Park et al. combined Ti_5_NbO_14_ nanosheets with Ag_2_CO_3_/Ag to construct Ag_2_CO_3_/Ag-Ti_5_NbO_14_ multicomponent nanohybrids, which exhibited a much more superior photodegradation activity under visible light in comparison to the unhybridized Ag_2_CO_3_ sample [23]. It highlighted that the construction of the heterojunction structure was beneficial to the retarded recombination rate of photogenerated electrons and holes, leading to the remarkable improvement of photodegradation activity.

In this work, we successfully fabricated 1D S-doped K_3_Ti_5_NbO_14_@TiO_2_ (STNT) core-shell heterostructured composites, showing an improved visible-light-driven photodegradation activity for the removal of methylene blue (MB) due to the combined effects of S doping and heterojunction. Furthermore, photo-electrochemistry analysis, photoluminescence (PL), and time-resolved transient PL decay spectra were employed to investigate the interfacial charge separation of the STNT composite. A possible photodegradation mechanism was also proposed. Consequently, this work will provide an insight into fabricating core-shell hetero-structured catalysts with a high photocatalytic performance.

## 2. Materials and Methods

K_3_Ti_5_NbO_14_ rod was synthesized based on our previous work with a detailed synthetic process as follows: A mixture of K_2_CO_3_ (33 mmol, an excess of 10%), TiO_2_ (100 mmol), and Nb_2_O_5_ (10 mmol) was fully ground and then heated at 1050 °C for 24 h with a heating rate of 5 °C/min.

The as-prepared K_3_Ti_5_NbO_14_ rod (2.0 g) was first well-dispersed in ethanol with ultrasonic treatment. After stirring for 12 h, different volumes (1, 2, 3, 4, and 5 mL) of titanium isopropoxide, Ti(O-*i*-Pr)_4_, were added into the above suspension and stirred for 12 h, respectively. This suspension was then exposed to the air so as to allow ethanol evaporation. Finally, 1.0 g of the obtained Ti precursor was mixed, finely milled with thiourea (2.0 g), and then heated at 500 °C for 5 h with a heating rate of 2 °C/min in air. The targeted samples of S-doped K_3_Ti_5_NbO_14_@TiO_2_ core-shell materials were denoted as STNT*x*, where *x* (*x* = 1 2, 3, 4, and 5) represent the added volume of Ti(O-*i*-Pr)_4_ in the precursors. For STNT-*x* (*x* = 1, 2, 3, 4, and 5), the corresponding loading amount of TiO2 is 26.7, 42.4, 52.3, 59.3, and 64.6 wt%, respectively. For comparison, the undoped samples of K_3_Ti_5_NbO_14_@TiO_2_ core-shell material (denoted as TNT*x*) and S-doped TiO_2_ (denoted as S-TiO_2_) were also prepared by a similar process only without the addition of thiourea and K_3_Ti_5_NbO_14_, respectively.

**Figure 1:**
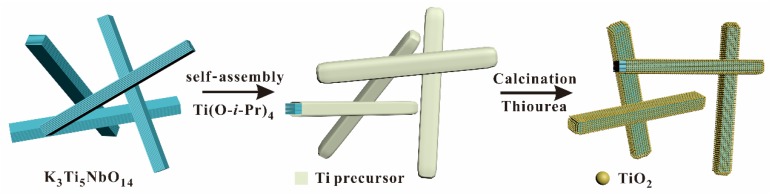
A schematic illustration for synthesizing S-doped K_3_Ti_5_NbO_14_@TiO_2_ core/shell materials.

The schematic illustration for synthesizing S-doped K_3_Ti_5_NbO_14_@TiO_2_ (STNT) core-shell materials is displayed in Figure 1. The bulk K_3_Ti_5_NbO_14_ was firstly mixed with Ti(O-*i*-Pr)_4_, leading to the full deposition of the Ti precursor on the surface of K_3_Ti_5_NbO_14_ by a re-assembly process. Then, the resulted mixture was heated in air in the presence of thiourea to obtain core-shell STNT, in which the layered structure of K_3_Ti_5_NbO_14_ was maintained and TiO_2_ nanoparticles (NPs) were uniformly formed on the surface of K_3_Ti_5_NbO_14_.

The characterization techniques and photocatalytic experimental process are supplied in the Supplementary Material.

## 3. Results

### 3.1. Structure and Morphology

**Figure 2:**
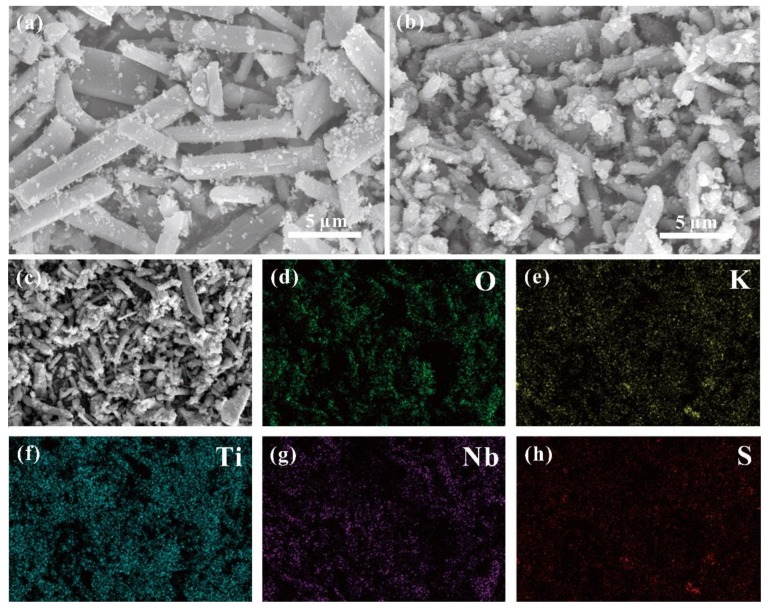
SEM images of (**a**) K_3_Ti_5_NbO_14_ and (**b**) S-doped K_3_Ti_5_NbO_14_@TiO_2_-3 (STNT3). (**c**–**h**) SEM-EDS mappings of STNT3 with the distribution of O, K, Ti, Nb, and S elements.

As shown in Figure 2a, layered K_3_Ti_5_NbO_14_, with a rod-like morphology, can be clearly observed, exhibiting a length of 5–15 µm. The choice for K_3_Ti_5_NbO_14_ rods as a starting material is due to the fact that anisotropically shaped rods show the decreased recombination rate of photogenerated electron−hole pairs [14,24]. For the STNT3 composite (Figure 2b), a rod-like structure is maintained while the surface became relatively rough with a somewhat decreased crystallinity relative to pure K_3_Ti_5_NbO_14_, owing to the deposition of TiO_2_ NPs on the surface of K_3_Ti_5_NbO_14_. Energy dispersive spectra (EDS) mapping images of STNT3 are displayed in Figure 2c–h. It can be clearly visible that the elements of O, K, Ti, Nb, and S are uniformly dispersed on the surface of STNT3, giving direct evidence for the introduction of the S element in STNT3. The corresponding elemental contents of STNTS are provided in Table S2, showing only 1.70 wt% of doped S in STNT3.

**Figure 3:**
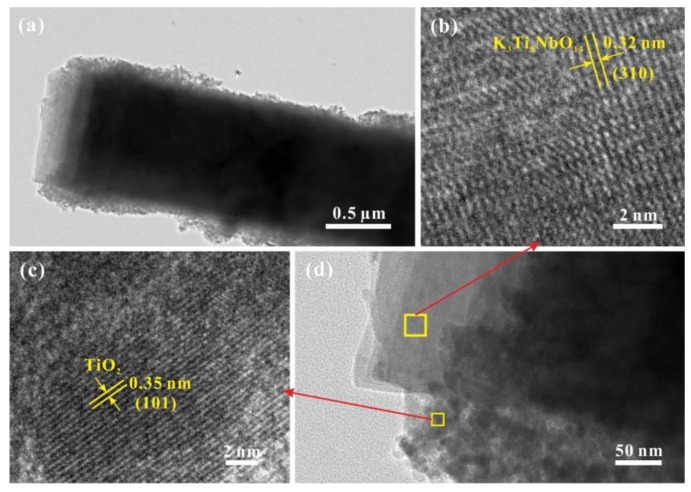
TEM (**a**) and HRTEM (**b**–**d**) images of STNT3.

Under TEM observation (Figure 3a), the sample of STNT3 shows a clear core-shell structure. Some overlapped sheets are observed on the edge, indicating the maintenance of a layered structure. Additionally, some nanoparticles, maybe TiO_2_ NPs, are formed on the surface of K_3_Ti_5_NbO_14_ rods. HRTEM image of STNT3 (Figure 3d) further confirm the formation of a core-shell structure. The enlarged HRTEM images of STNT3 (Figure 3b,c) show the clear lattice fringes with a respective value of 0.32 and 0.35 nm, which are in accordance with (310) facet of K_3_Ti_5_NbO_14_ and (101) facet of anatase TiO_2_ NPs based on the crystallographic symmetry, respectively. It indicates that K_3_Ti_5_NbO_14_@TiO_2_ core-shell materials are formed, in which TiO_2_ nanopartiocles (NPs) with a size of ~10–20 nm are deposited on the surface of K_3_Ti_5_NbO_14_ rods to form a nanoscale heterojunction structure between two components. This heterojunction structure is favorable for the separation of photogenerated electrons and holes [25,26]. In addition, the exposed reductive (101) facet of anatase TiO_2_ NPs has been regarded as a possible reservoir of photogenerated electrons for the reduction of O_2_ to produce •O_2_^−^, resulting in the promoted separation of charge carriers [27].

### 3.2. Powder X-Ray Diffraction Analysis

**Figure 4:**
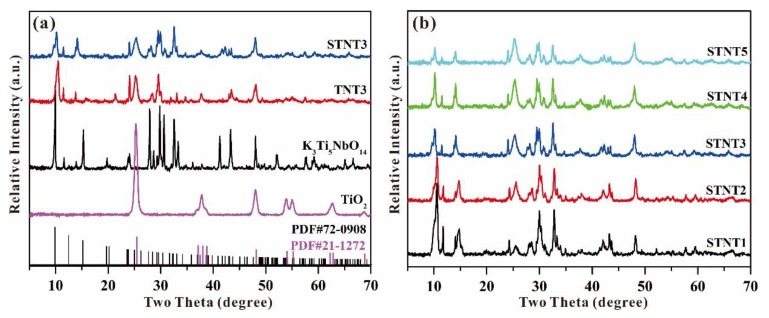
XRD patterns of (**a**) different samples, and (**b**) as-prepared STNT*x* (*x* = 1 2, 3, 4, and 5) composites.

The crystallinity of the obtained samples was characterized by XRD patterns and displayed in Figure 4a. The XRD pattern of K_3_Ti_5_NbO_14_ is in line with the published data (PDF, #72-0908). After heating the mixture of K_3_Ti_5_NbO_14_ and Ti(O-*i*-Pr)_4_, some new diffraction peaks with a decreased intensity can be obviously observed in TNT3 related to pure K_3_Ti_5_NbO_14_, indicating the formation of anatase TiO_2_ based on the published data (PDF, #21-1272). After further S doping, the sample of STNT3 shows similar characteristic peaks with TNT3. However, compared with pure K_3_Ti_5_NbO_14_, the characteristic (200) peak is almost unchanged in STNT3, while a slight shift toward a higher angle in TNT3, indicating the improvement of structural stability in STNT3 within the addition of thiourea.

As displayed in Figure 4b, when K_3_Ti_5_NbO_14_ was calcinated with different volumes of Ti(O-i-Pr)4, the resultant STNTx (x = 1 2, 3, 4 and 5) samples show similar XRD patterns, including two crystal phases of K_3_Ti_5_NbO_14_ and anatase TiO_2_. As the added volume of Ti(O-i-Pr)4 is increased, the peak intensity of STNTx is gradually decreased, due to the increased amount of TiO_2_ NPs shell on the surface of K_3_Ti_5_NbO_14_ rods. Furthermore, the crystal phases of K_3_Ti_5_NbO_14_ in all STNTx (x = 1 2, 3, 4 and 5) samples are well maintained, indicating that the introduction of TiO_2_ NPs and S doping have little impact on the crystal structure of K_3_Ti_5_NbO_14_.

### 3.3. UV-Vis Analysis

**Figure 5:**
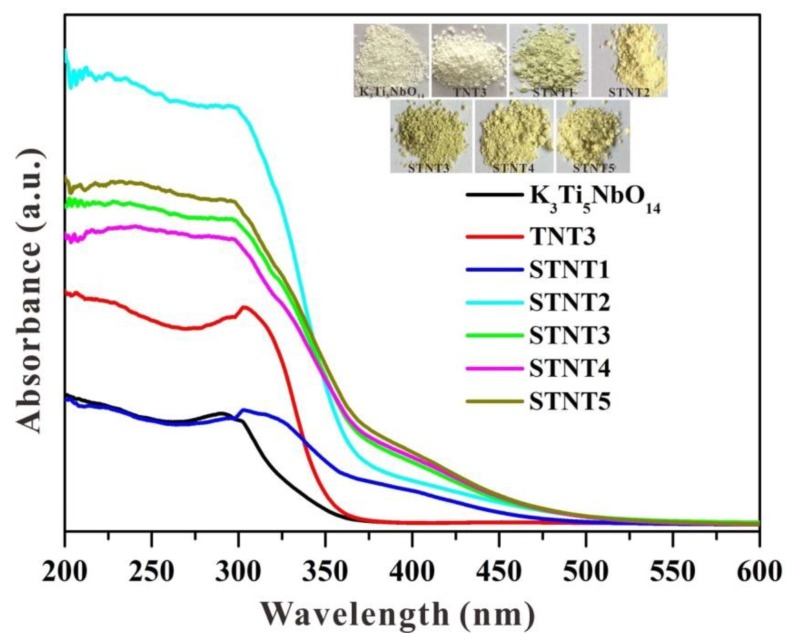
UV-visible diffuse reflectance spectra of as-prepared photocatalysts (Inset is the images of the corresponding samples).

UV-visible diffuse reflectance spectra (DRS) of K_3_Ti_5_NbO_14_, TNT3, and STNT composites are displayed in Figure 5. For pure K_3_Ti_5_NbO_14_ with a bandgap value of 3.82 eV (Figure S1a), only an absorption band in the UV-light region is visible, owing to the band-to-band transition rather than the transition from the impurity levels [28]. After combining with anatase TiO_2_ NPs, the light absorption in the UV-light region is profoundly enhanced in TNT3, which may be due to the formed heterojunction between two components by the electronic coupling and the possible quantum size effect [18,29,30]. After S doping, the absorption edges of all STNT composites show a prominent redshift in comparison with pure K_3_Ti_5_NbO_14_, confirming the enhanced visible-light absorption. Compared with samples from STNT1 to STNT3, the light absorption is almost unchanged from STNT3 to STNT5, indicating that an appropriate ratio between K_3_Ti_5_NbO_14_ and anatase TiO_2_ is essential for constructing heterojunction structure and thus achieving a broadened light absorption region. The light absorption of samples is consistent with their corresponding color changes from white to grey yellow.

### 3.4. XPS Analysis

**Figure 6:**
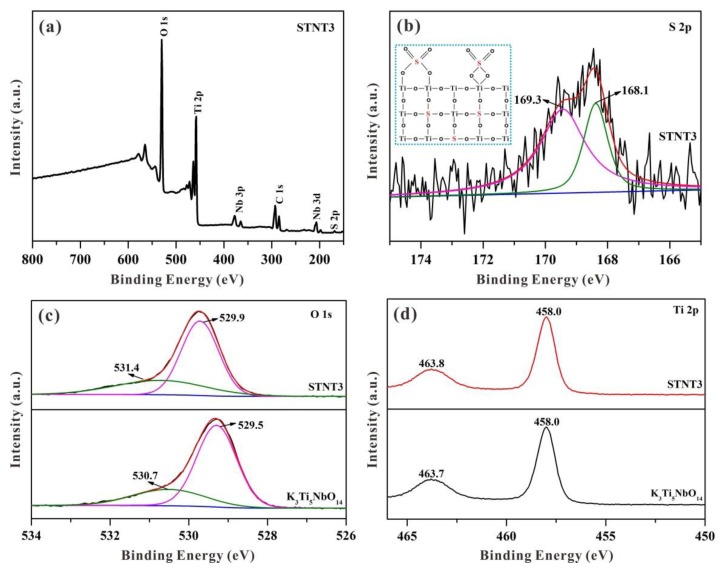
High-resolution XPS spectra for K_3_Ti_5_NbO_14_ and STNT3: (**a**) survey, (**b**) S 2p spectrum (inset shows two possible structures of the surface-adsorbed sulfate groups in STNT3), (**c**) O 1s, and (**d**) Ti 2p.

XPS spectra were employed to determine the chemical composition and atomic states of as-prepared samples. As displayed in Figure 6a, the survey spectrum of STNT3 includes C, O, Ti, Nb, and S elements, in which the carbon peak stems from adventitious hydrocarbon. As shown in Figure 6b, a broadened sulfur peak, at 168.5 eV, can be clearly observed in STNT3 due to the split sublevels of S 2p_3/2_ and S 2p_1/2_ states. The amount of doped S atoms was calculated to be approximately 1.81 at% by XPS. After fitting, two strong XPS peaks, at both 169.3 and 168.1 eV, are obtained with a separation of 1.20 eV owing to spin-orbit coupling [18]. These two strong peaks indicate that the element of doped S is to be in the form of S^6+^ (SO_4_^2−^ groups), resulting in the formation of Ti−O−S bonds and charge imbalance [31,32]. The substitute of Ti^4+^ by S^6+^ in the lattice of STNT3 is generally much more favorable than replacing O^2−^ with S^6+^ [33]. This doped S by the substitution of Ti^4+^ would be similar to that of metal-ion doping, creating intra-band-gap states below the conduction band (CB) position [19]. As the external surface of the K_3_Ti_5_NbO_14_ core is fully covered with anatase TiO_2_ NPs, the S element is primarily incorporated into the lattice of the TiO_2_ shell.

According to the above analysis, it is reasonable to propose two possible binding models between surfaced SO_4_^2−^ groups and anatase TiO_2_ based on the previous literature (Figure 6b) [31,34]. The surfaced SO_4_^2−^ in STNT3, driven by the heat treatment under atmospheric conditions, can be used as the efficient electron trapping center for promoting the separation of photogenerated electron–hole pairs. To achieve charge balance, the formed cationic STNT3 is probably favorable for neutralization by the adsorbed hydroxide ions (OH^−^_ads_), and thus capture the photogenerated holes to yield hydroxyl radicals (^•^OH) as the main active groups for the degradation of MB.

It can be seen in Figure 6c that the O 1s spectrum of K_3_Ti_5_NbO_14_ can be evaluated by two peaks at 529.5 and 530.7 eV, associated with 2-coordinated oxygen (O1) and 4-coordinated oxygen (O_2_), respectively [35]. In comparison with pure K_3_Ti_5_NbO_14_, O 1s spectrum of STNT3 shows similar two peaks with a positive shift. The main peak at 529.9 eV can be attributed to lattice oxygen, while the peak at 531.4 eV is ascribed to oxygen in sulfate. These changed O 1s species in STNT3, with higher binding energies, may be activated.

Two peaks at 458.0 (Ti 2p_3/2_) and 463.8 eV (Ti 2p_1/2_) in Figure 6d can be clearly visible in the Ti 2p spectrum of STNT3. These two peaks are quite similar to those in K_3_Ti_5_NbO_14_, which is attributed to the octahedrally coordinated Ti. Compared with K_3_Ti_5_NbO_14_, only a slight shift of Ti 2p is noticed maybe due to the interaction between Ti atoms and SO_4_^2−^ anions, leading to the change of electron density around the Ti atoms. Additionally, no shoulder peak, attributed to Ti^3+^ species, can be seen suggesting that the chemical state of the Ti atom is mainly in the form of Ti^4+^ in STNT3 [36].

### 3.5. Photocatalytic Activity

**Figure 7:**
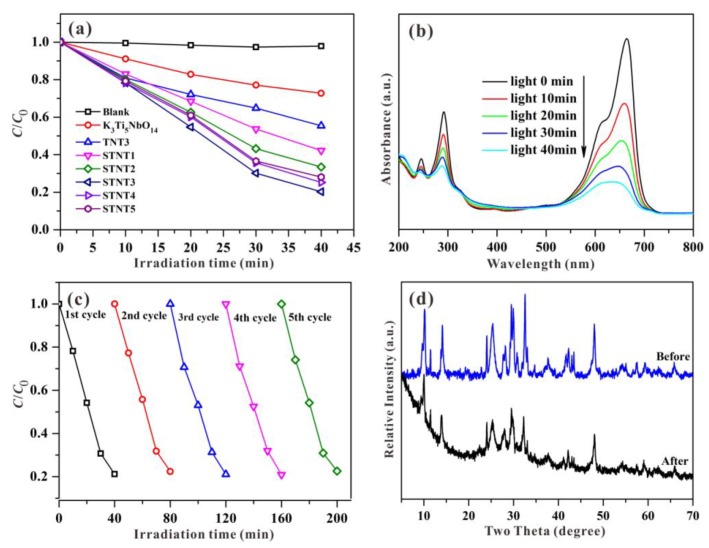
(**a**) Visible-light-driven photocatalytic degradation rate of MB solution over different samples. (**b**) UV-visible spectral changes, (**c**) recycling experiments, and (**d**) XRD patterns before and after five cycles over STNT3 for the photocatalytic degradation of MB.

To evaluate the photocatalytic performance, MB was chosen as a targeted pollutant with visible-light irradiation. As the irradiation time is increased, the reduced concentration of MB is displayed in Figure 7a. Based on the blank control experimental result, the simulated pollutant of MB exhibited a high stability and the neglect of self-photolysis. For bulk K_3_Ti_5_NbO_14_, approximately ~28% of the MB concentration can be decreased under visible light within 40 min, owing to self-sensitization oxidation [37]. Although, the wide-band-gap semiconductor of K_3_Ti_5_NbO_14_ itself cannot be excited under visible light, the targeted pollutant of MB can well absorb visible light to produce photogenerated electrons and then transferred into the CB edge of K_3_Ti_5_NbO_14_ owing to the potential difference, resulting in the visible-light-driven photocatalytic activity [24].

After combining with TiO_2_ NPs, the obtained sample of TNT3 exhibits the improved photodegradation activity for the removal of MB due to the formed heterojunction between K_3_Ti_5_NbO_14_ and TiO_2_, which is beneficial for the efficient spatial separation of photo-induced charge carriers [38,39,40]. After S doping, the photocatalytic activity of the resultant STNT3 is further enhanced in comparison to TNT3. It suggests that S doping can essentially tune the electric structure of TNT3, leading to the visible-light response and thus enhanced photocatalytic activity. Additionally, from Figure S3, the photocatalytic activity of STNT3 is higher than that of S-TiO_2_ NPs due to the role of heterojunction formation between K_3_Ti_5_NbO_14_ and TiO_2_.

By comparison, all STNT*x* (*x* = 1 2, 3, 4, and 5) composites exhibit the significantly improved visible-light-driven photocatalytic activities relative to K_3_Ti_5_NbO_14_, due to the synergistic effects of S doping and the formed heterojunction. As the added volume of Ti(O-*i*-Pr)_4_ is increased, the photocatalytic activities of STNT*x* composites are firstly increased and then decreased with the highest activity for STNT3. It suggests that an appropriate mass of added Ti(O-*i*-Pr)_4_ is necessary to fabricate the effective heterojunction structure between K_3_Ti_5_NbO_14_ and TiO_2_, which is beneficial for the achievement of excellent photocatalytic activity. Nevertheless, the surface of K_3_Ti_5_NbO_14_ will be fully covered by adding the excess of Ti(O-*i*-Pr)_4_, and thus the photo-induced electrons cannot be effectively transferred from the CB of S-TiO_2_ to the CB of K_3_Ti_5_NbO_14_ across the straddling band structure, resulting in reduced photocatalytic activity.

As displayed in Figure 7b, as the photocatalytic reaction time passes, the characteristic absorption bands of MB are profoundly decreased under visible light within STNT3. The reduction in absorbance may be due to the destruction of the molecular structure. After recycling five times (Figure 7c), the sample of STNT3 still shows a high photocatalytic activity, indicating the excellent visible-light-driven photocatalytic stability for the removal of MB. Additionally, the XRD pattern of STNT3, after recycling in Figure 7d, shows no obvious difference in comparison with the original STNT3, demonstrating a high structural stability of STNT3.

### 3.6. Electrochemical Analysis and PL Analysis

**Figure 8:**
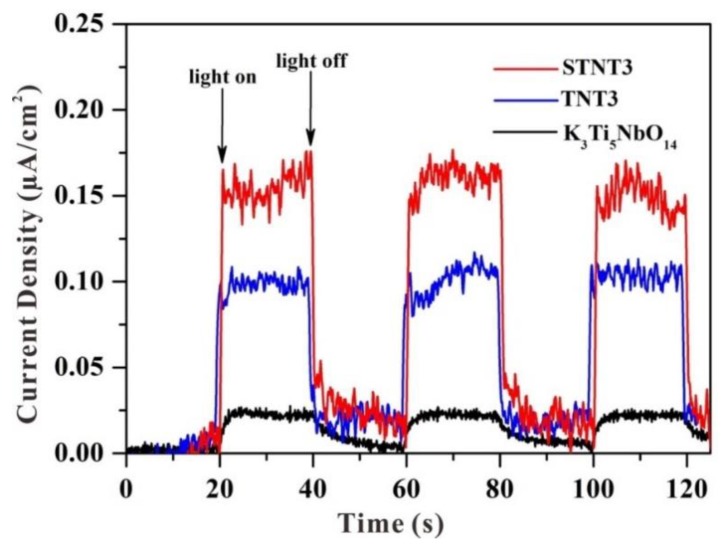
Transient photocurrent responses of different samples under simulated solar light irradiation.

The transient photocurrent responses of K_3_Ti_5_NbO_14_, TNT3, and STNT3 are displayed in Figure 8. The decay of photocurrent intensity demonstrates the recombination between a fraction of holes and electrons [41,42,43]. A relatively stable photocurrent response can be observed for all samples. The wide-band-gap K_3_Ti_5_NbO_14_ exhibits the lowest photocurrent intensity due to its large bandgap value. After combination with TiO_2_ NPs, the signal of TNT3 is greatly enhanced, indicating that the formation of the heterojunction between K_3_Ti_5_NbO_14_ and TiO_2_ is beneficial for the effective separation of photogenerated electron–hole pairs. After further S doping, the intensity of the obtained STNT3 is higher than that of TNT3, indicating the rapid charge transfer rate and high separation efficiency of STNT3.

**Figure 9:**
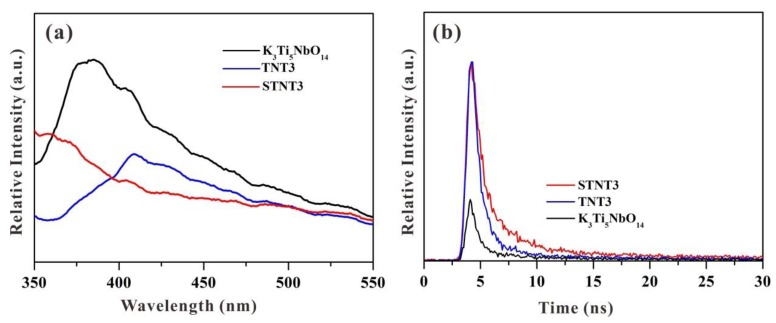
(**a**) Photoluminescence (PL) spectra with an excitation wavelength of 325 nm and (**b**) time-resolved transient PL decay spectra for K_3_Ti_5_NbO_14_, TNT3, and STNT3.

The photoluminescence (PL) spectroscopy was commonly applied in the photocatalytic field to evaluate the recombination rate of the photo-induced charge carriers [44,45]. Generally, the higher PL emission intensity indicates the severer recombination rate of photogenerated electrons and holes [46]. The PL spectra of K_3_Ti_5_NbO_14_, TNT3, and STNT3 are displayed in Figure 9a. A broad peak, at approximately 380 nm, can be clearly visible for bulk K_3_Ti_5_NbO_14_, owing to the band-gap recombination of photo-induced electron–hole pairs. After hybridizing with TiO_2_ NPs, the PL intensity of the as-prepared TNT3 is lower than that of K_3_Ti_5_NbO_14_, suggesting that the hybridization of TiO_2_ NPs can profoundly decrease the recombination rate of photo-induced election–hole pairs by forming a heterojunction structure. Compared with TNT3, the signal of STNT3 is largely suppressed, which is mainly due to the fact that S doping will increase the light-harvesting capacity and thus restrict the recombination rate of photo-generated electron–hole pairs. This phenomenon suggests that the recombination rate of photo-generated charge carriers is profoundly hindered by the synergistic effects of S doping and heterojunction formation, which is consistent with the photocatalytic results.

The separation process of photo-induced charge carriers was also investigated by time-resolved transient PL spectroscopy (Figure 9b) [47,48]. As the photocatalyst absorbs photons, electrons are stimulated to the excited state and then reach the thermal equilibrium state, and the excess energy will be released by the nonradiative and radiative decay processes [49]. Generally, the emission lifetime is associated with the separation rate of charge carriers [50]. The average emission lifetimes of bulk K_3_Ti_5_NbO_14_, TNT3, and STNT3 can be calculated to be 0.97 ns, 2.56 ns, and 4.75 ns, respectively. In comparison with K_3_Ti_5_NbO_14_, the emission lifetime of TNT3 is increased due to heterojunction formation, which is favorable for the efficient transfer of photogenerated charge carriers. After further S doping, the lifetime of STNT3 is restricted due to the increased amount of light absorption. Thus, the prolonged lifetime of STNT3 may be ascribed to the combined effects of S doping and heterojunction formation.

### 3.7. Photocatalytic Mechanism

**Figure 10:**
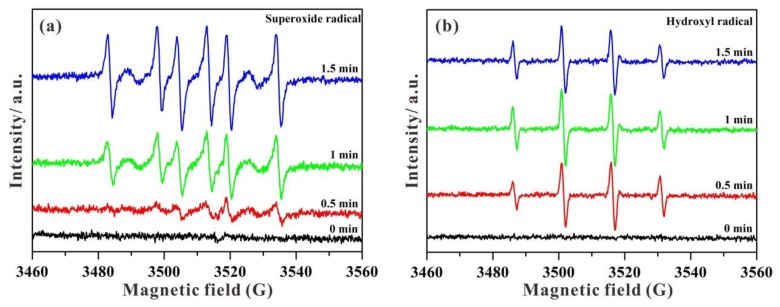
ESR spectra of STNT3 in aqueous solution before and after visible light irradiation: (**a**) DMPO-•O_2_^−^ and (**b**) DMPO-•OH.

Electron spin resonance (ESR) spectra were employed to investigate the existence of photogenerated •OH and •O_2_^−^ active species [51,52]. As displayed in Figure 10, no ESR signals of DMPO-•O_2_^−^ and DMPO-•OH can be visible in the dark. With increasing time under visible light irradiation, the intensities of DMPO-•O_2_^−^ signals are gradually enhanced (Figure 10a). Furthermore, some characteristic signals of DMPO-•OH can be clearly observed in Figure 10b with the relative intensities of 1:2:2:1. As a consequence, the ESR results confirm that active species of both •O_2_^−^ and •OH can be generated under visible light for photocatalytic MB degradation in STNT composites.

Combined with EM and XPS results, S element is primarily incorporated into the lattice of TiO_2_ shell to form S-doped TiO_2_ NPs as the surface of the K_3_Ti_5_NbO_14_ core is fully covered with anatase TiO_2_ shell. Pure K_3_Ti_5_NbO_14_ rods mainly act as electron transport carriers. As shown in Figure S1b, the bandgap value and valence band (VB) position are estimated to be 3.82 eV and +3.78 eV based on the combination of VB-XPS and DRS results, respectively. Combined with our previous work [18], the VB and CB positions of K_3_Ti_5_NbO_14_ and anatase TiO_2_ are estimated to be +3.78/−0.04 eV and +2.67/−0.53 eV, respectively. According to the Nernst Equation [53], the corresponding VB and CB potentials can be converted into +3.54/−0.28 V and +2.43/−0.77 V, respectively.

**Figure 11:**
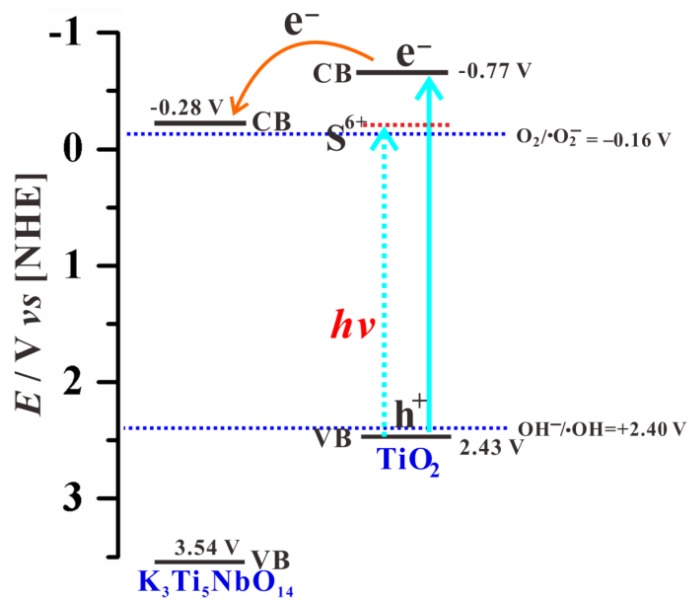
Energy levels of K_3_Ti_5_NbO_14_ and anatase TiO_2_ using a normal hydrogen electrode (NHE) as a reference at pH 7.

**Figure 12:**
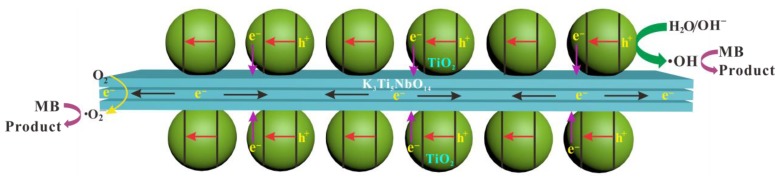
A schematic illustration of the STNT composite for the photodegradation of methylene blue (MB).

According to the above analyses, a possible photocatalytic mechanism for the removal of MB over STNT composite is proposed; the corresponding band energy levels and charge carrier behavior are schematically shown in Figure 11 and Figure 12. The fact that K_3_Ti_5_NbO_14_ and anatase TiO_2_ exhibit the analogous TiO_6_ octahedra structural features, which are favorable for the minimization of the lattice mismatch and thus promoting the deposition of anatase TiO_2_ NPs on the surface of K_3_Ti_5_NbO_14_ to form a closely contacted interface, leading to the nano-heterojunction formation. Under visible light, only S-doped TiO_2_ NPs in STNT composite can be excited to produce electrons and holes. The photogenerated electrons are transferred from VB to the intra-band-gap states created by S^6+^ and then to CB of TiO_2_. The accumulated electrons in CB of TiO_2_ (*E*_CB_ = −0.77 V) will quickly migrate to the CB of K_3_Ti_5_NbO_14_ (*E*_CB_ = −0.28 V) due to the potential difference, which can directly react with dissolved O_2_ to generate •O_2_^−^ (O_2_/•O_2_^−^, −0.16 V vs. NHE) [54]. Furthermore, as the VB potential of TiO_2_ (*E*_VB_ = +2.43 V) is positive than •OH/OH^−^ (+2.40 V vs. NHE) [54], the holes in the VB of TiO_2_ will react with OH^−^/H_2_O molecules to yield •OH radicals. Thus, MB aqueous solution may be oxidized by the combined effects of h^+^, •O_2_^−^ and •OH active species.

## 4. Conclusions

In summary, we successfully prepared 1D S-doped K_3_Ti_5_NbO_14_@TiO_2_ (STNT) core-shell rods heterostructured composites through a reassembly-calcination method, in which anatase TiO_2_ NPs were fully deposited on the external surface of K_3_Ti_5_NbO_14_ rods to form a tight close interface. The formed nanoscale heterojunction structure between K_3_Ti_5_NbO_14_ and TiO_2_ were favorable for the fostered separation of photogenerated electrons and holes. The S element is primarily incorporated into the lattice of TiO_2_, resulting in the formation of an intra-band-gap state below the CB position and thus the improved absorption of visible light. The resulting STNT composites showed an improved visible-light-driven photodegradation activity for the removal of MB, attributed to the combined effects of S doping and nanoscale heterojunction formation. The MB aqueous solution may be oxidized by the combined active species of h^+^, •O_2_^−^, and •OH. As the added amount of TiO_2_ NPs increased, the photocatalytic activities of STNT composites were firstly increased and then decreased with the optimal sample of STNT3. It is suggested that an appropriate mass between K_3_Ti_5_NbO_14_ and S-TiO_2_ NPs was beneficial for the achievement of the excellent photocatalytic activity. A possible photodegradation mechanism was proposed with a reasonable discussion. The present findings may provide an insight into designing and developing active photocatalysts with a core-shell structure toward sustainable environmental remediation.

## Supplementary Materials

The following are available online at https://www.mdpi.com/2079-4991/9/10/1503/s1, Figure S1. (a) (αhv)2 versus photo energy (hv) and (b) VB-XPS spectrum of pure K3Ti5NbO14. Figure S2. High-resolution XPS spectra for STNT3: (a) C 1s spectrum, and (b) N 1s spectrum. Figure S3. Visible-light-driven photocatalytic degradation rate of methylene blue (MB) solution over K3Ti5NbO14, S-TiO2 and STNT3. Table S1. Summary of optical power and power density. Table S2. Summary of elemental contents for S-doped K3Ti5NbO14@TiO2-3 (STNT3).

## References

[1] He R., Xu D., Cheng B., Yu J., Ho W. (2018). Review on nanoscale Bi-based photocatalysts. Nanoscale Horiz..

[2] Fu J., Yu J., Jiang C., Cheng B. (2018). g-C_3_N_4_-Based heterostructured photocatalysts. Adv. Energy Mater..

[3] Ren J., Ouyang S., Chen H., Umezawa N., Lu D., Wang D., Xu H., Ye J. (2015). Effective mineralization of organic dye under visible-light irradiation over electronic-structure-modulated Sn(Nb_1−*x*_Ta*_x_*)_2_O_6_ solid solutions. Appl. Catal. B.

[4] Li X., Yu J., Jaroniec M. (2016). Hierarchical photocatalysts. Chem. Soc. Rev..

[5] Zhong W., Shen S., Feng S., Lin Z., Wang Z., Fang B. (2018). Facile fabrication of alveolate Cu_2−*x*_Se microsheets as a new visible-light photocatalyst for discoloration of Rhodamine B. CrystEngComm.

[6] Chen X., Liu L., Huang F. (2015). Black titanium dioxide (TiO_2_) nanomaterials. Chem. Soc. Rev..

[7] Sun S., Song P., Cui J., Liang S. (2019). Amorphous TiO_2_ nanostructures: Synthesis, fundamental properties and photocatalytic applications. Catal. Sci. Technol..

[8] Guo Z., Wang Q., Shen T., Hou X., Kuang J., Liu W., Cao W. (2019). Synthesis of 3D CQDs/urchin-like and yolk-shell TiO_2_ hierarchical structure with enhanced photocatalytic properties. Ceram. Int..

[9] Oseghe E.O., Msagati T.A.M., Mamba B.B., Ofomaja A.E. (2019). An efficient and stable narrow bandgap carbon dot-brookite composite over other CD-TiO_2_ polymorphs in rhodamine B degradation under LED light. Ceram. Int..

[10] Xiong H., Wu L., Liu Y., Gao T., Li K., Long Y., Zhang R., Zhang L., Qiao Z.A., Huo Q. (2019). Controllable synthesis of mesoporous TiO_2_ polymorphs with tunable crystal structure for enhanced photocatalytic H_2_ production. Adv. Energy Mater..

[11] Liu C., Zhang C., Wang J., Xu Q., Chen X., Wang C., Xi X., Hou W. (2018). N-doped CsTi_2_NbO_7_@g-C_3_N_4_ core-shell nanobelts with enhanced visible light photocatalytic activity. Mater. Lett..

[12] Zhai Z., Hu C.H., Yang X.Y., Zhang L.H., Liu C., Fan Y.N., Hou W.H. (2012). Nitrogen-doped mesoporous nanohybrids of TiO_2_ nanoparticles and HTiNbO_5_ nanosheets with a high visible-light photocatalytic activity and a good biocompatibility. J. Mater. Chem..

[13] Zhai Z., Huang Y.C., Xu L., Yang X.Y., Hu C.H., Zhang L.H., Fan Y.N., Hou W.H. (2011). Thermostable nitrogen-doped HTiNbO_5_ nanosheets with a high visible-light photocatalytic activity. Nano Res..

[14] D’Arienzo M., Carbajo J., Bahamonde A., Crippa M., Polizzi S., Scotti R., Wahba L., Morazzoni F. (2011). Photogenerated defects in shape-controlled TiO_2_ anatase nanocrystals: A probe to evaluate the role of crystal facets in photocatalytic processes. J. Am. Chem. Soc..

[15] Suzuki H., Tomita O., Higashi M., Nakada A., Abe R. (2018). Improved visible-light activity of nitrogen-doped layered niobate photocatalysts by NH_3_-nitridation with KCl flux. Appl. Catal. B.

[16] Shown I., Samireddi S., Chang Y.C., Putikam R., Chang P.H., Sabbah A., Fu F.Y., Chen W.F., Wu C.I., Yu T.Y. (2018). Carbon-doped SnS_2_ nanostructure as a high-efficiency solar fuel catalyst under visible light. Nat. Commun..

[17] Ohno T., Akiyoshi M., Umebayashi T., Asai K., Mitsui T., Matsumura M. (2004). Preparation of S-doped TiO_2_ photocatalysts and their photocatalytic activities under visible light. Appl. Catal. A.

[18] Liu C., Han R., Ji H., Sun T., Zhao J., Chen N., Chen J., Guo X., Hou W., Ding W. (2016). S-doped mesoporous nanocomposite of HTiNbO_5_ nanosheets and TiO_2_ nanoparticles with enhanced visible light photocatalytic activity. Phys. Chem. Chem. Phys..

[19] Liu C., Liang J., Han R., Wang Y., Zhao J., Huang Q., Chen J., Hou W. (2015). S-doped Na_2_Ti_6_O_13_@TiO_2_ core-shell nanorods with enhanced visible light photocatalytic performance. Phys. Chem. Chem. Phys..

[20] Al-Keisy A., Ren L., Xu X., Hao W., Dou S.X., Du Y. (2018). Selective ferroelectric BiOI/Bi_4_Ti_3_O_12_ heterostructures for visible light-driven photocatalysis. J. Phys. Chem. C.

[21] Yang J., Liang Y., Li K., Yang G., Yin S. (2019). One-step low-temperature synthesis of 0D CeO_2_ quantum dots/2D BiOX (X = Cl, Br) nanoplates heterojunctions for highly boosting photo-oxidation and reduction ability. Appl. Catal. B.

[22] Liu C., Xu G., Zhu Y., Xu Q., Yu G., Hou H., Xu Q., Xi X., Hou W. (2018). In situ construction of layered K_3_Ti_5_NbO_14_/g-C_3_N_4_ composite for improving visible-light-driven photocatalytic performance. J. Mater. Sci..

[23] Park S., Lee J.M., Jo Y.K., Kim I.Y., Hwang S.J. (2014). A facile exfoliation-crystal growth route to multicomponent Ag_2_CO_3_/Ag-Ti_5_NbO_14_ nanohybrids with improved visible light photocatalytic activity. Dalton Trans..

[24] Liu C., Sun T., Wu L., Liang J., Huang Q., Chen J., Hou W. (2015). N-doped Na_2_Ti_6_O_13_@TiO_2_ core–shell nanobelts with exposed {1 0 1} anatase facets and enhanced visible light photocatalytic performance. Appl. Catal. B.

[25] Wang Y., Jiang W., Luo W., Chen X., Zhu Y. (2018). Ultrathin nanosheets g-C_3_N_4_@Bi_2_WO_6_ core-shell structure via low temperature reassembled strategy to promote photocatalytic activity. Appl. Catal. B.

[26] Wang Q., Wang W., Zhong L., Liu D., Cao X., Cui F. (2018). Oxygen vacancy-rich 2D/2D BiOCl-g-C_3_N_4_ ultrathin heterostructure nanosheets for enhanced visible-light-driven photocatalytic activity in environmental remediation. Appl. Catal. B.

[27] Liu G., Yang H.G., Pan J., Yang Y.Q., Lu G.Q., Cheng H.M. (2014). Titanium dioxide crystals with tailored facets. Chem. Rev..

[28] Xia Y., Liang S., Wu L., Wang X. (2019). Ultrasmall NiS decorated HNb_3_O_8_ nanosheeets as highly efficient photocatalyst for H_2_ evolution reaction. Catal. Today.

[29] Chen Z.J., Lin B.Z., Chen Y.L., Zhang K.Z., Li B., Zhu H. (2010). Pillaring and photocatalytic properties of mesoporous α-Fe_2_O_3_/titanate nanocomposites via an exfoliation and restacking route. J. Phys. Chem. Solids.

[30] Du G.H., Yu Y., Chen Q., Wang R.H., Zhou W., Peng L.M. (2003). Exfoliating KTiNbO_5_ particles into nanosheets. Chem. Phys. Lett..

[31] Yu J.C., Ho W., Yu J., Yip H., Wong P.K., Zhao J. (2005). Efficient visible-light-Induced photocatalytic disinfection on sulfur-doped nanocrystalline titania. Environ. Sci. Technol..

[32] Nasir M., Xi Z., Xing M., Zhang J., Chen F., Tian B., Bagwasi S. (2013). Study of synergistic effect of Ce- and S-codoping on the enhancement of visible-light photocatalytic activity of TiO_2_. J. Phys. Chem. C.

[33] El-Sheikh S.M., Zhang G., El-Hosainy H.M., Ismail A.A., O’Shea K.E., Falaras P., Kontos A.G., Dionysiou D.D. (2014). High performance sulfur, nitrogen and carbon doped mesoporous anatase–brookite TiO_2_ photocatalyst for the removal of microcystin-LR under visible light irradiation. J. Hazard. Mater..

[34] Devi L.G., Kavitha R. (2014). Enhanced photocatalytic activity of sulfur doped TiO_2_ for the decomposition of phenol: A new insight into the bulk and surface modification. Mater. Chem. Phys..

[35] Song Y., Wang H., Xiong J., Guo B., Liang S., Wu L. (2018). Photocatalytic hydrogen evolution over monolayer H_1.07_Ti_1.73_O_4_·H_2_O nanosheets: Roles of metal defects and greatly enhanced performances. Appl. Catal. B.

[36] Tan S., Xing Z., Zhang J., Li Z., Wu X., Cui J., Kuang J., Zhu Q., Zhou W. (2018). Ti^3+^-TiO_2_/g-C_3_N_4_ mesostructured nanosheets heterojunctions as efficient visible-light-driven photocatalysts. J. Catal..

[37] Liu C., Zhu H., Zhu Y., Dong P., Hou H., Xu Q., Chen X., Xi X., Hou W. (2018). Ordered layered N-doped KTiNbO_5_/g-C_3_N_4_ heterojunction with enhanced visible light photocatalytic activity. Appl. Catal. B.

[38] Wu Y., Wang H., Tu W., Liu Y., Tan Y.Z., Yuan X., Chew J.W. (2018). Quasi-polymeric construction of stable perovskite-type LaFeO_3_/g-C_3_N_4_ heterostructured photocatalyst for improved Z-scheme photocatalytic activity via solid p-n heterojunction interfacial effect. J. Hazard. Mater..

[39] Peng Z., Jiang Y., Wang X., Xu R.H., Xiao Y., Jing X., Zhang J., Liu Y., Ni L. (2019). Novel CdIn_2_S_4_ nano-octahedra/TiO_2_ hollow hybrid heterostructure: In-situ synthesis, synergistic effect and enhanced dual-functional photocatalytic activities. Ceram. Int..

[40] Yan Y., Yang M., Shi H., Wang C., Fan J., Liu E., Hu X. (2019). CuInS_2_ sensitized TiO_2_ for enhanced photodegradation and hydrogen production. Ceram. Int..

[41] Zhou H., Zhong S., Shen M., Yao Y. (2019). Composite soft template-assisted construction of a flower-like β-Bi_2_O_3_/Bi_2_O_2_CO_3_ heterojunction photocatalyst for the enhanced simulated sunlight photocatalytic degradation of tetracycline. Ceram. Int..

[42] Xu J., Yan X., Qi Y., Fu Y., Wang C., Wang L. (2019). Novel phosphidated MoS_2_ nanosheets modified CdS semiconductor for an efficient photocatalytic H_2_ evolution. Chem. Eng. J..

[43] Zhong W., Shen S., He M., Wang D., Wang Z., Lin Z., Tu W., Yu J. (2019). The pulsed laser-induced Schottky junction via in-situ forming Cd clusters on CdS surfaces toward efficient visible light-driven photocatalytic hydrogen evolution. Appl. Catal. B.

[44] Wei Z., Liu J., Fang W., Xu M., Qin Z., Jiang Z., Shangguan W. (2019). Photocatalytic hydrogen evolution with simultaneous antibiotic wastewater degradation via the visible-light-responsive bismuth spheres-g-C_3_N_4_ nanohybrid: Waste to energy insight. Chem. Eng. J..

[45] Zhao L., Qi Y., Song L., Ning S., Ouyang S., Xu H., Ye J. (2019). Solar-driven water–gas shift reaction over CuO*_x_*/Al_2_O_3_ with 1.1 % of light-to-energy storage. Angew. Chem. Int. Ed..

[46] Zhong W., Tu W., Feng S., Xu A. (2019). Photocatalytic H_2_ evolution on CdS nanoparticles by loading FeSe nanorods as co-catalyst under visible light irradiation. J. Alloys Compd..

[47] Li X., Yang G., Li S., Xiao N., Li N., Gao Y., Lv D., Ge L. (2010). Novel dual co-catalysts decorated Au@HCS@PdS hybrids with spatially separated charge carriers and enhanced photocatalytic hydrogen evolution activity. Chem. Eng. J..

[48] Zhao Z., Wu J., Zheng Y.Z., Li N., Li X., Tao X. (2019). Ni_3_C-Decorated MAPbI_3_ as visible-light photocatalyst for H_2_ evolution from HI splitting. ACS Catal..

[49] Cao S., Shen B., Tong T., Fu J., Yu J. (2018). 2D/2D Heterojunction of ultrathin MXene/Bi_2_WO_6_ nanosheets for improved photocatalytic CO_2_ reduction. Adv. Funct. Mater..

[50] Liang X., Zhang Y., Li D., Wen B., Jiang D., Chen M. (2019). 2D/2D BiOCl/K^+^Ca_2_Nb_3_O_10_^−^ heterostructure with Z-scheme charge carrier transfer pathways for tetracycline degradation under simulated solar light. Appl. Surf. Sci..

[51] Wang K., Li Y., Zhang G., Li J., Wu X. (2019). 0D Bi nanodots/2D Bi_3_NbO_7_ nanosheets heterojunctions for efficient visible light photocatalytic degradation of antibiotics: Enhanced molecular oxygen activation and mechanism insight. Appl. Catal. B.

[52] Liu C., Xu Q., Zhang Q., Zhu Y., Ji M., Tong Z., Hou W., Zhang Y., Xu J. (2019). Layered BiOBr/Ti_3_C_2_ MXene composite with improved visible-light photocatalytic activity. J. Mater. Sci..

[53] Yang X., Qian F., Wang Y., Li M., Lu J., Li Y., Bao M. (2017). Constructing a novel ternary composite (C_16_H_33_(CH_3_)_3_N)_4_W_10_O_32_/g-C_3_N_4_/rGO with enhanced visible-light-driven photocatalytic activity for degradation of dyes and phenol. Appl. Catal. B.

[54] Li Y., Lv K., Ho W., Dong F., Wu X., Xia Y. (2017). Hybridization of rutile TiO_2_ (rTiO_2_) with g-C_3_N_4_ quantum dots (CN QDs): An efficient visible-light-driven Z-scheme hybridized photocatalyst. Appl. Catal. B.

