# Supplementary material for: Core-Shell Heterostructured and Visible-Light-Driven Titanoniobate/TiO2 Composite for Boosting Photodegradation Performance
Source: Nanomaterials (Basel). 2019 Oct 22;9(10):1503. doi: 10.3390/nano9101503 (PMC6836207; doi:10.3390/nano9101503)
Supplement: Supplementary file 1 [file nanomaterials-09-01503-s001.pdf]

nanomaterials

Article

Core-Shell Heterostructured and Visible-Light-Driven Titanoniobate/TiO_2 Composite for Boosting Photodegradation Performance

Chao Liu [1,2], Xin Gao [1], Zitong Han [1], Yao Sun [1], Yue Feng [1], Guiyun Yu [3], Xinguo Xi [3,*], Qinfang Zhang [1,4,*], and Zhigang Zou [2]

[1] School of Materials Science and Engineering, Yancheng Institute of Technology, Yancheng, 224051, China.
[2] Eco-Materials and Renewable Energy Research Center (ERERC), College of Engineering and Applied Sciences, Nanjing University, Nanjing 210093, China
[3] School of Chemistry & Chemical Engineering, Yancheng Institute of Technology, Yancheng, 224051, China.
[4] Key Laboratory for Advanced Technology in Environmental Protection of Jiangsu Province, Yancheng Institute of Technology, Yancheng 224051, China.
* Correspondence: xxg@ycit.cn (X. X); qfangzhang@gmail.com (Q. Z); Tel. and Fax: (+86)-515-88298251 (X. X); (+86)-515-88298249 (Q. Z).

1. Experimental Section

1.1. Characterization Techniques

The crystal morphology of the as-prepared samples was investigated by field emission scanning electron microscopy (FE-SEM, JEOL JEM-6300F, Tokyo, Japan) and transmission electron microscopy (TEM, JEOL JEM-200CX, Tokyo, Japan). The EDS elemental mapping analysis, equipped with a FE-SEM machine, was taken on an energy dispersive X-ray spectrometer (EDS, JEOL JEM-6300F, Tokyo, Japan) in order to study the spatial elemental distribution. The phase composition of the obtained samples was investigated via X-ray powder diffraction (XRD) patterns using a Cu Kα radiation (λ = 1.5418 Å) and a scanning rate of 0.2°/s. UV–vis diffuse reflectance spectra (UV-vis DRS) were carried out on an UV–vis spectrophotometer (Shimadzu, UV-3600Plus, Kyoto, Japan) using $BaSO_4$ as a reference. X-ray photoelectron spectroscopy (XPS) measurement was taken on an Thermo Fisher Scientific spectrometer (New York, NY, USA) equipped with a hemispherical electron analyzer using an Al Kα ($h\nu$ = 1486.6 eV) X-ray source. All binding energies were referenced to the C 1s peak (284.6 eV) from surface adventitious carbon. Photoluminescence (PL) spectra of those samples were recorded on a Hitachi-F4500 with an excitation of 325 nm. The time-resolved PL (TR-PL) decay spectra were recorded by an F900 fluorescence spectrophotometer (Hitachi, Tokyo, Japan) with an excitation wavelength of 375 nm. The photocurrent was carried out in a standard three-electrode system on CHI-660D electrochemical station with a 500 W Xe arc lamp as the light source. The electrolyte solution was Na_2SO_4 aqueous solution (0.1 M). The platinum wire and Ag(s)/AgCl(s) were used as the counter and reference electrodes, respectively.

1.2. Photocatalytic Degradation Reaction Tests

To evaluate photocatalytic activity, 0.1 g of catalyst was added into MB aqueous solution (2 mg/L) and then placed in the dark for 1 h to gain the adsorption-desorption equilibrium with a pH value of ~5.7 for the obtained suspension. A 300 W Xe lamp ($\lambda \geq$ 420 nm) (Aulight, Beiing, China), with a UV light filter film, was used as a light source. The corresponding optical power and power density of Xe lamp were shown in Table S1. At given time intervals, approximately 4 mL of the reaction suspension was sampled and then filtered by a 0.45 μm filter. The maximum absorbance of MB at 664 nm was recorded on a UV-visible spectrophotometer. The degradation efficiency was determined based on the value of Ct/C0, where C0 and Ct are the concentrations at initial period and time t, respectively.

Table S1. Summary of optical power and power density.

d [a] (cm)	Optical Power (mW)	Optical Power Density (mW/cm^2)
1	459	601
2	421	566
3	403	552
4	381	515
5	370	508
6	355	479
7	331	446
8	301	413

[a] *d* means the distance betweem thin film filter and the probe of CEL-NP2000. The starting upper and lowest liquid surfaces of dyes solution are about 3 and 9 cm apart from filter in the photocatalytic tests, respectively.

Table S2. Summary of elemental contents for S-doped $K_3Ti_5NbO_{14}$@TiO_2-3 (STNT3).

	K	O	Ti	Nb	S
Weight percentage (wt.%)	10.45	36.90	21.42	29.55	1.70
Atomic percentage (at.%)	2.89	71.68	13.90	9.89	1.64

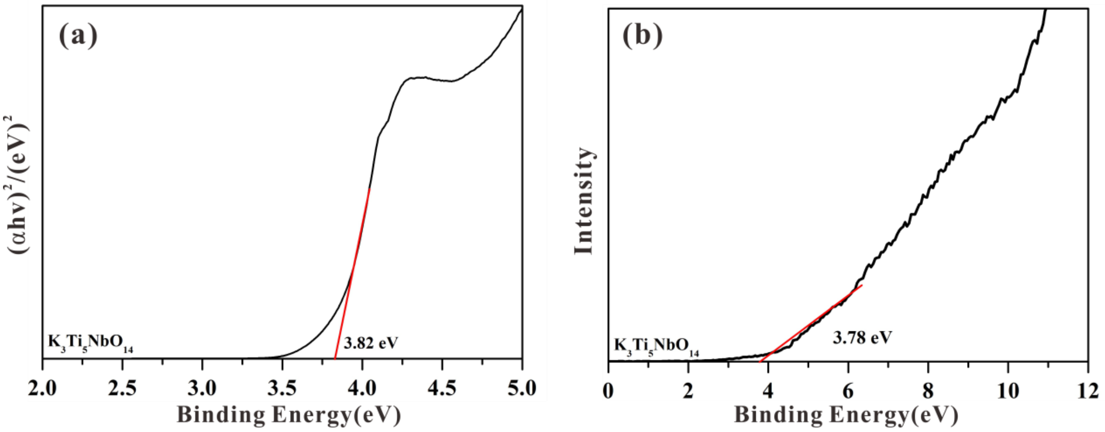

Figure S1. (**a**) $(\alpha hv)^2$ versus photo energy (hv) and (**b**) VB-XPS spectrum of pure $K_3Ti_5NbO_{14}$.

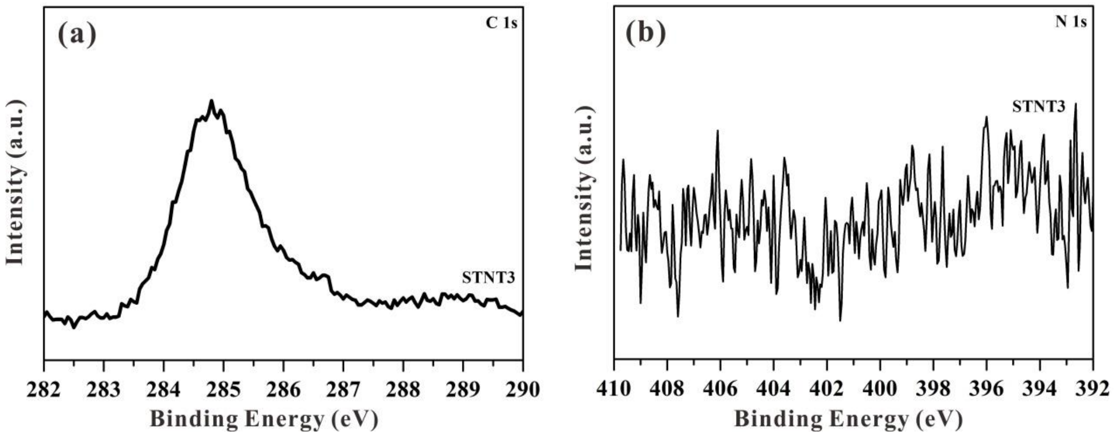

Figure S2. High-resolution XPS spectra for STNT3: (**a**) C 1s spectrum, and (**b**) N 1s spectrum.

From C1s XPS of STNT3, the main carbon peak at 284.6 eV can be observed due to the adventitious hydrocarbon from the XPS measurement. No obvious peak at ~288.0 eV, ascribed to sp2-bonded carbon of N = C−N of g-C_3N_4 was observed, indicating that no carbon

nitride like material was formed on the surface of $K_3Ti_5NbO_{14}$ in this work [1,2]. Additionally, No obvious N signal can be also visible.

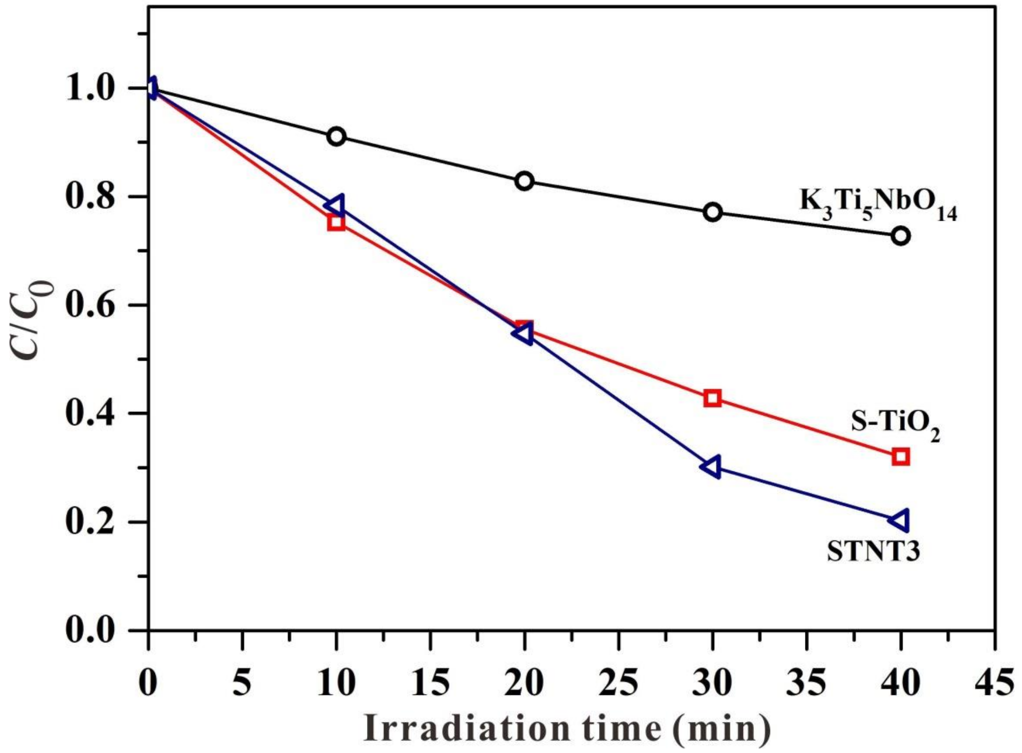

Figure S3. Visible-light-driven photocatalytic degradation rate of methylene blue (MB) solution over $K_3Ti_5NbO_{14}$, S-TiO_2 and STNT3.

References

1. Wang, K.; Li, Q.; Liu B.; Cheng, B.; Ho, W.; Yu, J. Sulfur-doped g-C_3N_4 with enhanced photocatalytic CO_2-reduction performance, *Appl. Catal. B* **2015**, *176–177*, 44–52.
2. Xiao, J.; Xie, Y.; Nawaz, F.; Wang, Y.; Du, P.; Cao, H.; Dramatic coupling of visible light with ozone on honeycomb-like porous g-C_3N_4 towards superior oxidation of water pollutants. *Appl. Catal. B* **2016**, *183*, 417–425.

© 2019 by the authors. Submitted for possible open access publication under the terms and conditions of the Creative Commons Attribution (CC BY) license (http://creativecommons.org/licenses/by/4.0/).
